# Exploring the Feasibility of Bidirectional Control of Beta Oscillatory Power in Healthy Controls as a Potential Intervention for Parkinson’s Disease Movement Impairment

**DOI:** 10.3390/s24165107

**Published:** 2024-08-06

**Authors:** Krithika Anil, Giorgio Ganis, Jennifer A. Freeman, Jonathan Marsden, Stephen D. Hall

**Affiliations:** 1 School of Health Professions, University of Plymouth, Drake Circus, Plymouth PL4 8AA, UK; jonathan.marsden@plymouth.ac.uk; 2 Brain Research and Imaging Centre, Faculty of Health, University of Plymouth, Research Way, Plymouth PL6 8BU, UK; giorgio.ganis@plymouth.ac.uk (G.G.); stephen.d.hall@plymouth.ac.uk (S.D.H.); 3 School of Psychology, University of Plymouth, Drake Circus, Plymouth PL4 8AA, UK; 4 Peninsula Allied Health Centre, School of Health Professions, University of Plymouth, Derriford Road, Plymouth PL6 8BH, UK

**Keywords:** neurofeedback, EEG, motor control, Parkinson’s disease, reaction time

## Abstract

Neurofeedback (NF) is a promising intervention for improvements in motor performance in Parkinson’s disease. This NF pilot study in healthy participants aimed to achieve the following: (1) determine participants’ ability to bi-directionally modulate sensorimotor beta power and (2) determine the effect of NF on movement performance. A real-time EEG-NF protocol was used to train participants to increase and decrease their individual motor cortex beta power amplitude, using a within-subject double-blind sham-controlled approach. Movement was assessed using a Go/No-go task. Participants completed the NASA Task Load Index and provided verbal feedback of the NF task difficulty. All 17 participants (median age = 38 (19–65); 10 females) reliably reduced sensorimotor beta power. No participant could reliably increase their beta activity. Participants reported that the NF task was challenging, particularly increasing beta. A modest but significant increase in reaction time correlated with a reduction in beta power only in the real condition. Findings suggest that beta power control difficulty varies by modulation direction, affecting participant perceptions. A correlation between beta power reduction and reaction times only in the real condition suggests that intentional beta power reduction may shorten reaction times. Future research should examine the minimum beta threshold for meaningful motor improvements, and the relationship between EEG mechanisms and NF learning to optimise NF outcomes.

## 1. Introduction

Parkinson’s disease is a progressive neurological disorder that affects 1–2 in every 1000 individuals in the general population [1] and primarily disrupts motor functioning, including slowing (bradykinesia) and freezing (akinesia) of movement. Motor symptoms in Parkinson’s are believed to be driven by dopaminergic neurodegeneration in the basal ganglia (BG [2]). This is considered to result in abnormal neural firing in the subthalamic nucleus (STN) and globus pallidus internus, with increased power in the population firing at a beta frequency (15–30 Hz [3]) that dysregulates effective movement control [4]. Currently, the primary treatments are medications that increase dopaminergic drive and provide symptomatic relief. Accordingly, symptomatic improvement from medications is accompanied by a reduction in beta frequency connectivity [4], with a similar beta reduction accompanying effective deep brain stimulation of BG nuclei [5,6]. Importantly, the attenuation of medication efficacy over time and increased incidence of adverse side effects, such as dyskinesias [7,8], introduce additional movement challenges and markedly impact a patient’s quality of life.

Oscillations have long been established as critical markers of communication within and between networks, with putative roles for cortical brain rhythms in the coordination of effective function across cognitive and behavioural domains. In conditions such as Parkinson’s, these rhythms are enhanced and seem to underlie the bradykinesia and akinesia [9,10]. Importantly, while aberrant beta is likely driven by BG, it is also observed in non-invasive measurement of motor cortex, where it is also directly reflects symptom severity [11,12]. Interestingly, some research suggests that beta power is diminished compared to controls in later-stage Parkinson’s, with some restoration of synchrony afforded by medication [13].

In the sensorimotor cortex of healthy participants, self-paced movement is characterised by beta desynchronisation in the pre-movement period up to two seconds prior to movement initiation [14]. Following this, whether self-paced or externally cued, there is a further reduction in beta power, referred to as movement-related beta desynchronisation (MRBD [15]). Following movement termination, an increase in beta oscillatory power above the pre-movement baseline amplitude is observed [16], a phenomenon referred to as post-movement beta rebound (PMBR [17]). These robustly observed patterns of beta synchrony demonstrate the importance of dynamic bidirectional oscillatory modulation in achieving effective movement.

Recent research suggests that neurofeedback (NF) using cortical oscillations to control visual stimuli [18] is a potentially valuable method that enables an individual to non-invasively modulate their neural network properties in real-time. This approach has promising implications for improving oscillation-dependent motor function in Parkinson’s. However, a recent systematic review by our group [19] highlighted a range of inconsistencies and limitations in previously applied methods, providing recommendations for improving upon the inconsistent outcomes reported in the NF literature. Accordingly, this study aims to contribute to the establishment of effective NF approaches that provide individuals with robust control over neural network properties with functional implications. Here, we focus on the bidirectional modulation of beta power, which has powerful implications as adjunct or alternative therapeutic interventions for Parkinson’s and related disorders.

In this pilot study, we applied an EEG-based NF approach with two objectives. Firstly, we aimed to determine the ability of NF to provide bidirectional control of sensorimotor beta oscillations. Secondly, we aimed to determine the effect of NF and oscillatory control on movement performance. As a secondary aim, we captured participants’ perspective of task feasibility to contextualise the real-world potential and refine future approaches.

### Related Work

Several scalp-EEG-based NF protocols have been examined in the past few decades, showing that individuals can change their brain activity. Fumuro et al. [20] showed that 40–45% of their participants successfully controlled their CZ pre-motor potentials, while Kasahara et al. [21] showed that 58–65% of their participants successfully controlled their sensorimotor beta power (9.5–12.5 Hz). More recently, He et al. [22] showed that participants suppressed their sensorimotor beta bursts in their real condition but not in their sham condition. Similarly, Cook et al. [23] has shown in their case study that an individual with Parkinson’s successfully controlled their sensorimotor rhythm. However, He et al. [22] is the only study that incorporated a sham-controlled approach and included a movement performance measure in recent years, showing that reaction time improvements correlated with NF training. Many studies do not include a motor performance measure to examine the associate between NF performance and movement, as shown by our recent systematic review [24]. There is a clear need for further sham-controlled neurofeedback studies.

Previous studies have highlighted that not all participants are successful in controlling their EEG activity. Alkoby et al. [25] reported in their general NF review that between 16 and 57% of participants were unsuccessful in controlling their brain. Our systematic review [19] on Parkinson’s NF studies found that success rates varied between 40 and 100%, and several studies did not report any success rates. Understanding the NF learning mechanism may increase NF performance success rates that will support the optimisation of NF as an intervention for Parkinson’s. Kadosh and Staunton [26] reviewed 16 studies that examined behavioural predictors of NF performance but were unable to identify a clear performance predictor. They concluded that general behavioural concepts, such as mood, are likely to influence individuals’ effort. Anil et al. [24] similarly found that emotions experienced during NF training impacted performance, where feelings of discontent with NF performance and being mentally tired were associated with being unsuccessful. These emotions were influenced by the mental workload and perceived difficulty of the NF task, suggesting that individuals may adjust their efforts in an NF task according to the emotions. Therefore, it is important to understand how individuals engage with NF to identify NF learning mechanisms. Currently, only one Parkinson’s NF study, that we are aware of, has examined NF learning. Han et al. [27] showed that using an NF protocol based on imaginary movement strategies led to successful sensorimotor rhythm control and improved movement in people with Parkinson’s, suggesting that specific strategies may be more useful in NF than others. However, this was not compared to the use of other mental strategies, and it is therefore difficult to state whether the outcome was specific to the use of imagined movements. Due to the lack of learning information in this field, this pilot study aimed to explore perceived difficulty of the NF task. In addition, the NF instructions and related observations were recorded to lay the foundations for future NF learning research. This is provided as a Supplementary Materials (SM1-Neurofeedback Instructions) as it was deemed outside the scope of this pilot study.

## 2. Methods

### 2.1. Participants

Nineteen right-handed participants were recruited to the study, resulting in a complete usable dataset for seventeen participants (10F, 7M), with a median age of thirty-eight (range: 19–65). Participants were recruited from the University of Plymouth via email advertisement using convenience sampling. The exclusion criteria included neurological and upper-limb musculoskeletal conditions and medications impacting neural function.

Ethical approval was provided by the Ethics Committee from the Faculty of Health within the University of Plymouth (19/20-1322). All participants were provided with an information sheet and had the opportunity to ask questions prior to the start of the study. Written informed consent was taken from all participants before starting the study. Participants were reminded of their right to withdraw from the study at any time without reason and were provided appropriate breaks through the study procedure.

### 2.2. Study Design and Protocol

This pilot study used a double-blind, sham-controlled, within-subject design in which all participants completed a real-NF and a sham-NF session. Sessions were completed on different days, with a counterbalanced order assigned randomly by a computer. The study was unblinded only when the EEG analysis was completed. Experimenter’s blinding was achieved by creating a file at the beginning of the study with a list of random sham/NF pair entries that was automatically read in at runtime. This file was created by one of the authors (GG) and was not visible to the experimenter (KA).

In each visit, participants first completed a calibration task consisting of 32 Go/No-go trials (50/50 ratio) to identify the individually specific beta frequency maximum (fmax_β_) at C3, which was referenced to Cz. Electrodes were placed according to the standard 10–20 system [28], with a focus on contralateral sensorimotor cortex C3 which was chosen as the only electrode based on a review of previous Parkinson’s NF studies [19]. This frequency, ±5 Hz (clipped at 14 Hz and 31 Hz for the lower and upper limits, respectively), was used to define the beta range (R_β_) for all subsequent NF and data processing (see EEG Methods). This processing of the beta range minimised any impact of changes in individual EEG activity between the real and sham visits.

The Go/No-go task, used for both calibration and NF sessions, required participants to generate or withhold a dominant-hand index-finger button-press response at imperative cue (IC) onset. The Go/No-go task was chosen as it is a standard movement control task used to track speeded response times as well as response inhibition over time and used in previous studies examining Parkinson’s movement [29,30].

The stimuli consisted of the following: (i) a fixation cross at the center of the screen for 2000 ms; (ii) replaced by a yellow bar on screen for 4250–5000 ms, with 750 ms random jitter, directing participants to prepare to move; iii) this was then replaced by an imperative cue, either a green ‘Go’ rectangle (71%, 84 trial) or red ‘No-go’ rectangle (29%, 24 trials), on screen for 4000 ms. These timings were chosen based on the protocol by He et al. [22]. Participants were instructed to respond as quickly as possible on the Go trials and not to respond on the No-go trials. They were given feedback if they were too slow on the Go trials (>800 ms), if their response time was shorter than 100 ms, or if they responded incorrectly. Correct Go trials were defined as trials on which participants responded within 100 and 800 ms following the imperative stimulus.

**Figure 1:**
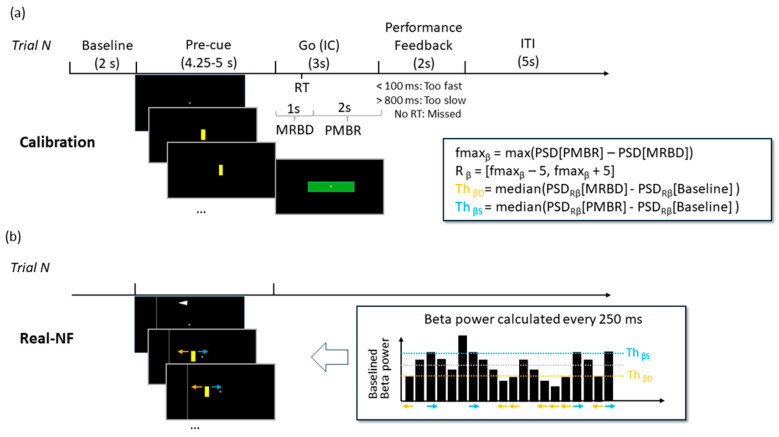
Diagram summarising the timing and computations for a Go trial during the calibration (**a**) and real-NF phases (**b**). Note that the timeline is not to scale. For the calibration phase, a baseline period (black screen) was followed by the pre-cue (a stationary yellow bar at the center of the screen) and then by the imperative cue (IC, a green rectangle on Go trials and a red rectangle on No-go trials). Next, participants pressed the response key as fast as possible (or withheld response on No-go trials), they would receive feedback on their performance before waiting for the next trial. For the NF phase, everything was the same with the exception that a white arrow on top of the display (pointing left in this example) appeared at the end of the baseline to indicate in which direction to move the pre-cue. The pre-cue was the same yellow bar, but its motion was controlled by the participant’s beta power.

In the subsequent training and NF experiment phases, the yellow bar was presented with a direction arrow to indicate the direction that the bar should be moved. In the real-NF experiment, a rightward arrow and bar movement required the participant to increase beta power compared to baseline (fixation cross), while a leftward arrow required a reduction in beta power (see Figure 1). In the sham-NF experiment, the bar position was not coupled to the current beta power. Instead, a replay of each participant’s calibration phase EEG was used to provide a realistic representation. The NF experiment included a total of 84 Go trials and 24 No-go trials (plus 4 practice trials), provided in six blocks of 5 min, interleaved by a 5 min break. During the breaks, verbal feedback was obtained for the following two questions: “how easy or difficult did you find moving the bar to the left?” and “how easy or difficult did you find moving the bar to the right?” In addition, the NASA Task Load Index [31], scored from 0 to 100 (low to high), was used to obtain the participants’ perceived NF task workload. This provided qualitative and quantitative information on task feasibility, informing the neurofeedback learning context. Analysis of these behavioural data is at the end of this Methods section. Before the calibration phase and after the NF experiment, we collected 3 min of eyes-open resting baseline. The process of instructing participants and related observations were also recorded, which can be seen in the Supplementary Document (SM1-Neurofeedback Instructions).

Power spectral density (PSD) was calculated every 250 ms on the last 500 ms of the incoming C3-Cz EEG stream. The calibration phase was used to calculate fmax_β_ (maximum beta frequency) and R_β_ (beta range) for every participant. Fmax_β_ was computed offline by subtracting the PSD from the MRBD (0–1 s post-IC) and PMBR (1–3 s post-IC) periods, using the nominal beta range (14–31 Hz), and by finding the maximum. R_β_, defined as fmax_β_ ± 5 Hz, was then used to calculate the sync/desync beta thresholds (th_βD_ and th_βS_) to be used during the NF phase. These thresholds were calculated by subtracting PSD(baseline) from PSD(MRBD) and PSD(PMBR) for each trial, using R_β_ as the beta range, and by computing the median value across trials (separately, for MRBD and PMBR).

For the real-NF phase, beta power was calculated every 250 ms as for the calibration phase (using R_β_ as beta range). The two thresholds, applied to baselined beta power, were used to control the direction of movement of the yellow bar at each 250 ms time step during the pre-cue period (left for beta desynchronisation and right for beta synchronisation).

For the sham-NF phase, the EEG data saved from the calibration phase controlled the movement of the yellow bar rather than the ongoing EEG.

### 2.3. EEG Methods

#### 2.3.1. Recordings

EEG was recorded using a 32-channel actiCHamp Plus system (Brain Vision, Garner, NC, USA). Data were sampled at 500 Hz. No online filters were used and the EEG data + event streams were saved in a .xdf format, reference-free, via the Python actiChamp connector (Brain Products, Gilching, Germany [32]) and LabRecorder (Lab Streaming Layer [33]) applications. Incoming EEG data were processed in real-time with custom MATLAB (MathWorks, Natick, MA, USA) software. Power spectrum density (PSD) was computed on the detrended data, weighted by a symmetric Hamming window (length = 250). PSD was calculated as the squared absolute value of the power spectrum output for the defined frequency using the MATLAB FFT function.

#### 2.3.2. Calibration

**Figure 2:**
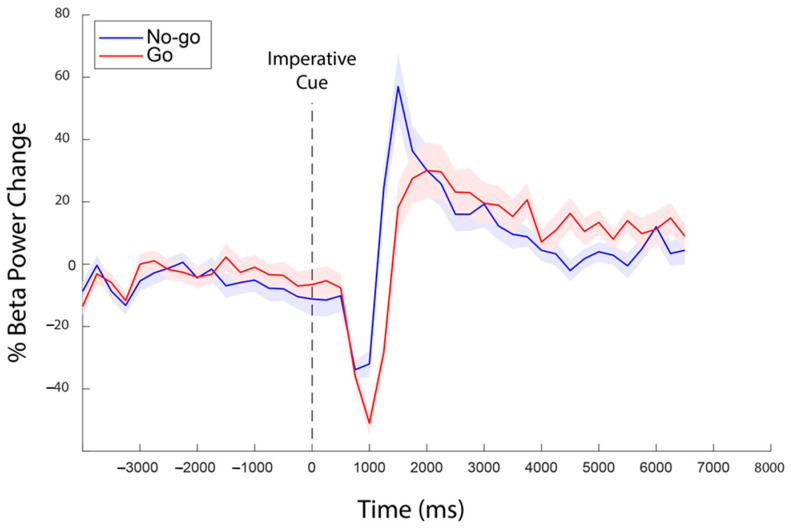
Averaged beta pattern across participants during the Go/No-go task of the calibration stage. Shaded bands indicate the standard error of the mean for each condition. Data showed here are time-locked to IC, and averages were computed from the saved beta power time courses calculated every 250 ms.

The calibration stage had two aims. The first aim was to confirm the presence of a resting-state beta band (14–31 Hz) signal and event-related beta desynchronisation (ERD) in the contralateral sensorimotor cortex, as measured from electrode C3. This was achieved by computing the mean power spectral density (PSD) of the baseline (eyes open) to confirm a peak in the beta range and then computing the mean power in the 14–31 Hz range over the time course of the correct Go Trials (1 s following IC onset) to confirm the presence of an ERD following the imperative cue onset. The same computation carried out between 1 and 3 s after IC onset confirmed the presence of a post-motor beta rebound (PMBR). The second aim was to calculate peak beta frequency (fmaxβ) and beta range (Rβ) for each participant, as well as beta power thresholds to use during the NF phase. Fmaxβ was computed offline by subtracting the PSD from the MRBD (0–1 s post-IC) and PMBR (1–3 s post-IC) periods using the nominal beta range (14–31 Hz) and by finding the frequency of the maximum difference. Rβ was defined as the frequency range from (fmaxβ − 5 Hz) to (fmaxβ + 5 Hz) and was used to calculate the sync/desync beta thresholds (th_βD_ and th_βS_) for the NF phase. These thresholds were calculated by subtracting PSD(baseline) from PSD(MRBD) and PSD(PMBR) for each trial, using Rβ as beta range, and by computing the median value across trials (separately, for MRBD and PMBR). To ensure that the online computation resulted in the expected beta patterns, the computed beta power timeseries were time-locked (i.e., synchronised in time) to trial onset and outliers (values greater than 3 scaled median absolute deviations away from the median: 1.4826*median[abs[x-median[x]]]) were replaced by linearly interpolating non-outlier entries. Figure 2 below shows the expected pattern, confirming that the NF software is identifying appropriate EEG activity. The raw EEG data were also analysed, as described below in the section titled “Analysis of Neurofeedback and Performance”.

#### 2.3.3. Neurofeedback Presentation

Visual stimuli were generated and controlled in real-time with Psychtoolbox [34] plugin for Matlab. For the real-NF session, the central starting position of the yellow bar position (Figure 1) on each trial was associated with baseline beta (BL_β_) power, defined as mean PSD of R_β_ in the 2 s preceding pre-cue onset. During each trial, the relative change (D_β_) in beta was computed in real-time as the mean of the present 500 ms window minus BL_β_, and it was updated every 250 ms. The position of the bar on screen was determined from D_β_ with a leftward movement of 20 pixels in any window where D_β_ < *th_βD_* and a rightward movement of 20 pixels in any window where D_β_ > *th_βs_*. If *th_βD_* ≤ D_β_ ≤ *th_βs_* in any window, the yellow bar would step back towards the center by 10 pixels. This design was intended to motivate participants to maintain engagement with the task throughout the trial. The position of the yellow bar was reset at the beginning of each trial. As described above, in the sham-NF session, the movement of the yellow bar was determined using the same algorithm, but replaying the data saved during the calibration phase for this purpose. Thus, there was no meaningful relationship between the position of the bar and the current R_β_ power. Each session took approximately 2.5 h and was completed on two separate days within one week. The 2.5 h are inclusive of breaks, where participants were regularly offered breaks throughout the session with instructions to take a break as long as they needed before continuing with the session.

### 2.4. Analysis

#### 2.4.1. Neurofeedback and Performance

EEG data were analysed offline using the EEGLab [35] plugin for MATLAB. Data from the real-NF and sham-NF sessions were epoched into the trials based upon the pre-cue onset (for NF analysis) and imperative cue marker (Go/No-go analysis). These epochs included two seconds prior to the cue onset and four seconds after the cue onset. The epochs were visually inspected for artifacts, and noisy trials were manually rejected. Subsequently, epochs were band-passed filtered (basic FIR filter new) based on the individual frequency ranges identified during the calibration stage (14–31 Hz). A notch filter was deemed unnecessary as the band-pass thresholds were below the main frequency interference (50/60 Hz). A time–frequency analysis was conducted to analyse the NF trials using the short-time Fourier transform that used a Hanning window with a size of 2 s and a 50% overlap. The power spectral density was computed and averaged across the ERD and ERS trials (i.e., decreasing and increasing beta trials respectively). Offline analysis also examined relative beta, which was calculated as the absolute power of beta during an NF trial (measured 4 s after cue onset) relative to the absolute power of beta during the baseline period (measured 2 s prior to cue onset).

#### 2.4.2. Perceived Neurofeedback Task Difficulty and Workload

The verbal responses regarding the difficulty of moving the bar to the left or right were analysed using content analysis [36]; this is a qualitative method that identifies patterned meaning across a dataset, using codes to identify common “themes” that summarise the main points explicitly presented in the data and counting them to describe their frequency. Themes were categorised according to group condition (i.e., real or sham). The NASA Task Load Index scores were analysed descriptively with mean and standard deviation.

## 3. Results

Data from two participants were excluded due to noise and non-completion, providing a total of 17 repeated measure datasets. In the remaining datasets, an analysis of the beta power confirmed the expected profile of baseline beta power, with MRBD and PMBR observed during movement execution and completion, respectively (see Figure 2). Analysis confirmed fmax_β_ within the 14–31 Hz band for all participants (mean 21 Hz, SD 6 Hz), with corresponding individual R_β_ defined accordingly.

**Figure 3:**
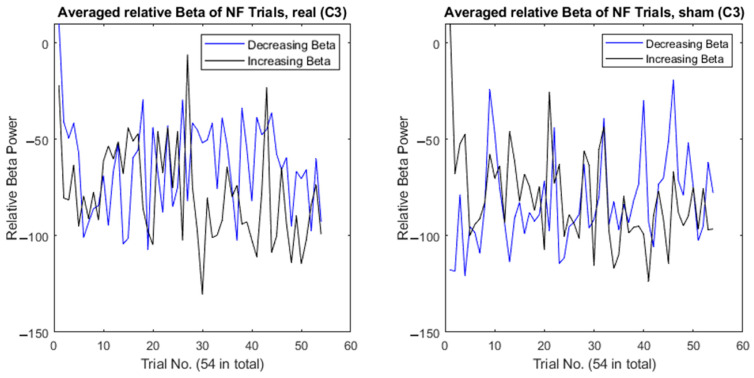
Comparison of beta power changes during neurofeedback training within a session. Each figure compares participants’ performance at attempting to decrease beta (i.e., blue line) and increase beta (i.e., black line). The left figure shows the comparison in the “real” condition and the right figure shows the comparison in the “sham” condition. The *x*-axis represents the averaged beta power across participants at each neurofeedback trial. The *y*-axis represents the averaged relative beta power, calculated as the absolute power of beta during an NF trial (measured 4 s after cue onset) relative to the absolute power of beta during the baseline period (measured 2 s prior to cue onset).

**Figure 4:**
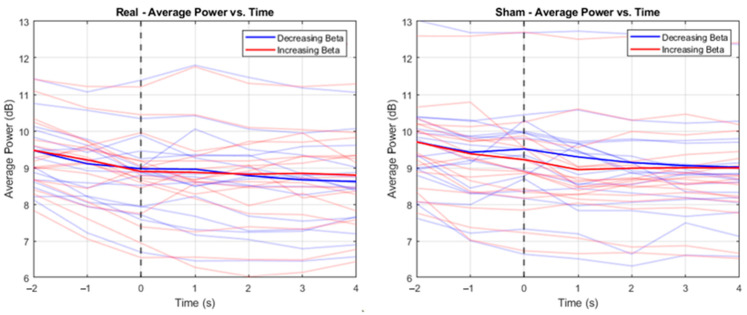
Time–frequency beta plot showing 2 s prior to neurofeedback stimuli onset and 4 s of the neurofeedback trial; dashed vertical line indicates onset of the yellow bar, i.e., the neurofeedback stimulus. Each figure compares participants’ performance at attempting to decrease beta (i.e., blue line) and increase beta (i.e., red line). The left figure shows the comparison in the “real” condition and the right figure shows the comparison in the “sham” condition. The *x*-axis represents beta power at each time point within a trial, where 0 represents the neurofeedback stimuli onset. The *y*-axis represents beta averaged across trials within a session.

Figure 3 shows how the relative beta power changed throughout the NF training averaged across participants, with a comparison between the real (decreasing beta = mean −100.98, SD 15.84; increasing beta = mean −100.16, SD 11.56) and sham (decreasing beta = mean −92.21, SD 16.08; increasing beta = mean −103.07, SD 9.71) conditions. Regardless of the instructed condition or direction of beta change (i.e., decrease or increase beta power), participants decreased beta during the NF period. This can be seen in Figure 4, which shows how beta decreased from onset of the NF stimulus, again regardless of the condition or required direction of beta change. This suggests that achieving an increase in beta power compared to baseline is particularly challenging. Wilcoxon signed-rank tests were conducted (Table 1 and Table 2) comparing beta power at each timepoint from Figure 4, which showed significant differences between decreasing and increasing beta in the sham condition at −1 s, 0 s, and 1 s (see Table 2). Another Wilcoxon signed-rank test showed a significant difference between the real and sham conditions when decreasing beta at 0 s (Z = −2.17, *p* = 0.030). No other significant differences were found.

Table 3 shows the outcomes of the Go/No-go task. Few errors were made during the Go and the No-go trials, and there is a negligible difference between the reaction times for the Go trials between conditions. The correlations between the Go/No-go outcomes and changes in relative beta were calculated, and they are included in the Supplementary Materials (SM2-Further Go/No-go Details). All correlations were small; however, there seemed to be a difference between the “left” (i.e., decreasing beta power) real condition versus all other conditions (mean r = 0.191 versus all other mean r < 0.1). Therefore, an ANOVA was undertaken to further investigate this relationship.

**Figure 5:**
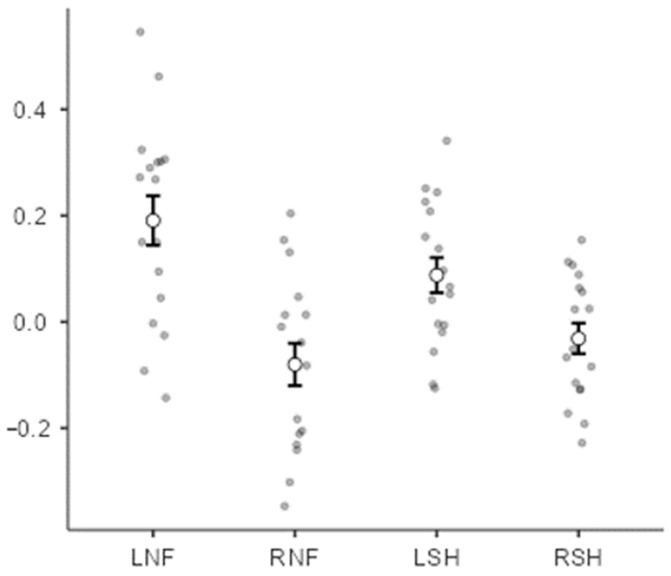
Comparisons of conditions showing the association between Go reaction times and relative EEG beta power. Each dot is a participant. LNF = “Left” neurofeedback trial (i.e., decreasing beta); RNF = “Right” neurofeedback trial (i.e., increasing beta); LSH = “Left” sham trial (i.e., decreasing beta); RSH = “Right” sham trial (i.e., increasing beta).

A one-way repeated measures ANOVA was conducted to examine the association between the Go reaction times and relative EEG beta power with the four conditions as levels (Figure 5). A significant difference was found between the conditions (F(3,48) = 10.5, *p* < 0.001). Post hoc contrasts corrected for multiple comparisons (Tukey’s method) showed that the correlation for the ‘left-real’ condition was higher than for the ‘right-real’ and ‘right-sham’ conditions, t(16) = 4.11, *p* = 0.004, and t(16) = 4.51, *p* = 0.002, respectively. However, it was not significantly higher than for the left sham condition, t(16) = 1.61, *p* = 0.403. The correlation for the right real condition was lower than for the left sham condition, t(16) = −3.43, but it did not differ from that of the right sham condition, t(16) = −1.06, *p* = 0.715.

### Perceived Neurofeedback Task Difficulty and Workload

Qualitative analysis of verbal responses identified the following themes:

- “Real” Condition

1. Moving the bar to the left was easy (*n* = 11, 65%).
2. Moving the bar to the right was hard (*n* = 16, 94%).
3. The task became harder as time went on (*n* = 10, 59%).

- “Sham” Condition

1. The task was generally hard (*n* = 12, 71%).
2. The task was a mix of easy and hard (*n* = 5, 29%).

The “real” condition theme indicated that participants found moving the bar to the left (i.e., decreasing beta) easy, while moving the bar to the right (i.e., increasing beta) was challenging. Gradually, the task became harder as time went on, indicating task fatigue or gradual disinterest in the task. The “sham” condition themes indicate that participants’ task difficulty perceptions were not related to the direction of the bar.

The NASA Task Load mean scores were 61.57 (SD = 14.35) and 65.57 (SD = 11.43) for the “real” condition and “sham” condition, respectively, indicating that both conditions had a high workload with negligible difference between them.

## 4. Discussion

This study showed that participants were only able to reliably decrease their sensorimotor beta power during the NF task. This is reflected in the qualitative responses, where participants consistently reported that decreasing beta power was “easy” while increasing beta power was a challenge, which may explain the NF performance outcomes. Participants reported this difference in difficulty between moving the bar to the left (“easy”) and to the right (“hard”) only for the “real” condition. During the “sham” condition, controlling the bar in either direction was typically regarded as difficult. This perception persisted despite no significant differences in NF performance between the real or sham conditions.

We also demonstrate a significant association between faster reaction times and a decrease in beta power in the “real” condition. The modest effect observed may reflect the comparatively small reduction in reaction time observed. Alternatively, it may indicate that the magnitude of beta power reduction was insufficient to produce a meaningful improvement in reaction time, or indeed that beta power is not causally related to movement initiation [37]. This finding is consistent with previous findings that show a positive correlation between cortical beta and faster reaction times [22,38], showcasing that altering movement-related EEG activity can impact movement. The specific cortical beta target differs in the previous studies, for example, He et al. [22] investigated beta bursts rather than beta power amplitude, suggesting that cortical beta as a whole is important to movement rather a specific feature of beta. Further exploration of this interaction is needed to understand these observations and potential implications for impacting movement outcomes and the related underlying EEG mechanism.

The observation that the significant correlation between beta power and reaction time only occurs in the “real” condition is of particular interest. The finding that spontaneously reduced beta in the sham condition does not correspond to an increase in reaction time indicates that reaction time may shorten only if there is an intentional reduction in motor cortical beta power. This finding further suggests that the beta frequency activity in the sensorimotor cortex is composed of multiple overlapping signals, that serve functionally separate purposes. There may also be a relation between this finding and the finding that participants reported this condition to be “easy” compared to other conditions. However, this finding may also be a consequence of greater variance in the sham condition, potentially impacting effect size. This implies a connection between the perception of the NF performance and the reaction time, which should be further investigated. This is of critical importance when considering the advancement of BCI approaches toward facilitation of a specific functional purpose.

Comparison of these results with existing evidence in Parkinson’s requires caution, due to the limited availability of research examining non-invasive EEG-based NF in Parkinson’s patients, as indicated by our systematic review [19]. The few papers published since this review have either targeted a different oscillatory rhythm (such as alpha [39]) or addressed different movement outcome measures [23]. The study by Cook et al. [23] is a single-case study that similarly demonstrated that decreasing beta power was possible with NF training but had an unclear impact on movement outcomes. This underscores both the potential application for movement disorders with a known cortical oscillatory association and the need for large-scale systematic exploration following proposed repeatable methods [19]. We suggest that a parallel stream of research exploring the relationship between more diverse EEG activity markers and the subcomponents of movement performance is required to optimise NF protocols with functionally targeted biomarkers.

However, the results observed here appear consistent with implanted electrode NF studies measuring beta signals from the sensorimotor cortex [40] and STN [41] of Parkinson’s patients. DBS research shows that bidirectional beta modulation is achievable but with greater reliability in reducing than increasing synchronous power [41]. Our study is further consistent with observed improvement in movement speed accompanying reduced beta power [42].

The present study focuses only on the beta frequency signatures in the contralateral sensorimotor cortex, based upon the implications for the amplitude of this signal in movement efficacy in Parkinson’s. However, it is important to note that extending NF approaches to include a broader frequency range and a wider array of electrodes may enable more reliable control and identify further therapeutic targets for NF.

Moreover, the behavioural and cognitive context of the NF training process appears to be an important aspect that requires careful examination. This study shows that participants had different perceptions of difficulty for the real and sham conditions, suggesting that learning criteria are critically important in NF performance, consistent with previous research examining the impact of mental strategies [24,27,43,44] or self-efficacy [24] on NF performance. Han et al. [27] showed that imaginary movement strategies led to successful sensorimotor rhythm control and improved movement in people with Parkinson’s disease, and they concluded that the use of imagined movements may improve factors such as spatial attention and mental imagery in people with Parkinson’s disease. The use of specific mental strategies may support NF training by reducing the potential impact of perceived difficulty, thereby leading to an improved NF training regime. We propose that a systematic examination of the factors that influence NF performance is required to optimise the performance of EEG-NF as an effective intervention.

The present study provides some valuable insights into the opportunities and considerations of EEG-NF approaches. While the sample size presents a limit to the generalisability of the findings, it is important to note that an effective NF approach is one that is effective at the individual level, rather than relying upon group effects. Reflecting on the protocol, we suggest that while the Go/No-go task is a valuable measure of performance, it is likely that interleaving the task disrupted the training process. Similarly, the inclusion of a bidirectional task, as opposed to unidirectional (increase or decrease beta) may have introduced a demand that was disruptive to the process. Finally, a systematic expansion of the qualitative approach, for example, using audio-recorded data, may inform better training methods.

## 5. Conclusions

This study presents an examination of bidirectional beta control and the impact upon behavioural performance, using a robust double-blinded sham approach. Understanding both the EEG mechanisms and behavioural aspects of NF training is crucial for optimising its real-world applications. Our findings indicate that beta power control varies in difficulty based on modulation direction, and participants’ perceptions of NF can shift according to training conditions. Future research should delve into these aspects to fully grasp their implications for NF performance. By engaging in interdisciplinary research that combines EEG mechanisms and behaviour, the NF field can progress and significantly benefit individuals with Parkinson’s disease.

## Figures and Tables

**Table 1:** Wilcoxon signed-rank test comparing beta power at each timepoint in the “real” condition.

	T1 (−2 s)	T2 (−1 s)	T3 (0 s)	T4 (1 s)	T5 (2 s)	T6 (3 s)	T7 (4 s)
	Decreasing beta condition						
Mean	8.85	8.14	7.89	7.87	7.56	7.36	7.29
SD	2.00	1.96	2.21	2.60	2.33	2.17	2.07
	Increasing beta condition						
Mean	8.86	8.34	7.76	7.71	7.62	7.66	7.58
SD	2.19	2.09	2.22	2.55	2.32	2.31	2.26

**Table 2:** Wilcoxon signed-rank test comparing beta power at each timepoint in the “sham” condition.

	T1 (−2 s)	T2 (−1 s) ^a^	T3 (0 s) ^b^	T4 (1 s) ^c^	T5 (2 s)	T6 (3 s)	T7 (4 s)
	*Decreasing beta condition*						
*Mean*	9.24	8.62	8.87	8.45	8.20	8.12	8.04
*SD*	3.29	3.12	3.16	3.27	3.20	2.92	2.91
	*Increasing beta condition*						
*Mean*	9.19	8.46	8.32	7.90	7.97	8.00	8.01
*SD*	2.84	3.00	3.18	3.06	3.12	2.88	2.99

Significant difference (*p* < 0.05, uncorrected for multiple comparisons) between the decreasing beta and increasing beta conditions: ^a^ Z = −2.07, *p* = 0.039. ^b^ Z = −2.69, *p* = 0.007. ^c^ Z = −2.28, *p* = 0.023.

**Table 3:** Averaged data for Go/No-go reaction times per condition.

**Average Reaction Times—Go Trials (All Errors Taken Out)**		
*Trial*	*Bar Direction*	*Average Reaction Time (s)*
Real	Left	0.489
Real	Right	0.493
Sham	Left	0.494
Sham	Right	0.494
**Average Errors Made—Go Trials**		
*Trial*	*Bar Direction*	*Average Count*
Real	Left	0.056
Real	Right	0.041
Sham	Left	0.027
Sham	Right	0.020
**Average Errors Made—No-go Trials**		
*Trial*	*Bar Direction*	*Average Count*
Real	Left	0.074
Real	Right	0.074
Sham	Left	0.029
Sham	Right	0.078
**Average Timings for Errors—Go Trials (Errors refer to “too slow” reactions; complete “misses” taken out)**		
*Trial*	*Bar Direction*	*Average Reaction Time (s)*
Real	Left	0.960
Real	Right	0.976
Sham	Left	0.962
Sham	Right	0.969
**Average Timings for Errors—No-go Trials**		
*Trial*	*Bar Direction*	*Average Reaction Time (s)*
Real	Left	0.477
Real	Right	0.485
Sham	Left	0.395
Sham	Right	0.410

## Data Availability

Data available in a publicly accessible repository via the University of Plymouth depository Pure.

## Supplementary Materials

The following supporting information can be downloaded at https://www.mdpi.com/article/10.3390/s24165107/s1, Document S1: SM1-Neurofeedback Instructions, Document S2: SM2- Further Go/No-go Details.
