# Supplementary material for: Exploring the Feasibility of Bidirectional Control of Beta Oscillatory Power in Healthy Controls as a Potential Intervention for Parkinson’s Disease Movement Impairment
Source: Sensors (Basel). 2024 Aug 6;24(16):5107. doi: 10.3390/s24165107 (PMC11358931; doi:10.3390/s24165107)
Supplement: Supplementary file 1 [file sensors-24-05107-s001.zip › SM1-Neurofeedback Instructions.pdf]

Supplementary Materials 1
Neurofeedback Instructions to Participants

Instructions

Task Difficulty

Prior to the neurofeedback training, the researcher explained the difficulty of the task so that participants would not be disheartened and give up on the task due to any perceived failures. The instructions were the following:

"Neurofeedback is a difficult task. We do not expect you to move the bar all the way to the goal post every time. Neurofeedback is a skill. Like with any skill, it improves with practice. Therefore, even small movements in the right direction will be regarded as a successful performance."

Mental Strategies

Participants were instructed to only use mental strategies and to avoid moving their body to try move the yellow bar on the screen. The researcher then let participants become familiar with the neurofeedback training during the first 5-minute block before suggesting strategies that may move the bar. Strategies were only suggested if participants requested them. The instructions were the following:

"Neurofeedback can be an intuitive task. This means sometimes participants cannot describe what they are doing to make the bar move but they are still successful. Therefore, I would like you to try the first neurofeedback block without any suggestions and see if you can come up with a strategy yourself. After the first block, I will ask you whether you would like any suggestions that may help you. These suggestions are based on what has been reported by participants in previous research. They may or may not help you move the bar as everyone's successful strategy is different."

Observations

The researcher encouraged participants throughout the study, and noted that some instructions were more helpful to participants than others. These observations were not systematically recorded, but are noted in this supplementary file as insights to lay the foundation for future examinations of neurofeedback learning.

1. Suggesting mental states were more useful to participants than specific mental strategies. For example, suggesting a "relaxed state of mind" was more useful than suggesting to "imaging relaxing on a warm beach". These states were noted to be a relaxed state, a stressed state, a focused state, or an emotional state.
2. A layman explanation of the targeted EEG activity seemed to be helpful for participants. The researcher briefly explained what the beta power represented and what behaviours were generally related to increasing and decreasing beta power. Participants seemed to "get" this explanation and were immediately able to come up with mental strategies themselves.
3. A layman explanation of the neurofeedback trial also seemed to be helpful for participants. The researcher explained that the "break" between the neurofeedback trials was used to calculate the EEG threshold to move the bar. This helped participants to "strategise" their approach to the neurofeedback task by attempting to "go back to a neutral state" between the neurofeedback trials to ensure the thresholds were not too difficult for them. However, participants admitted the break between the trials were short and they were not always able to "get back to neutral" in time.

The above observations are revolved around understanding neurofeedback and EEG processes at a lay level, and suggests that cognition plays an important role in neurofeedback learning. This would be an interesting topic to investigate as it will have implications in the design and implementation of neurofeedback protocols for optimal outcomes.
