# Supplementary material for: Exploring the Feasibility of Bidirectional Control of Beta Oscillatory Power in Healthy Controls as a Potential Intervention for Parkinson’s Disease Movement Impairment
Source: Sensors (Basel). 2024 Aug 6;24(16):5107. doi: 10.3390/s24165107 (PMC11358931; doi:10.3390/s24165107)
Supplement: Supplementary file 1 [file sensors-24-05107-s001.zip › SM2-Further Go No-go Details.pdf]

Supplementary Materials 2
Further Details on the Go/No-go Outcomes

Table 1 Average correlation between Go reaction times and relative EEG beta power across participants; all errors taken out

Real or Sham?	Beta Direction	Average Correlation
Real	Decreasing	0.191
Real	Increasing	-0.080
Sham	Decreasing	0.088
Sham	Increasing	-0.031

Table 2 Correlation between Go/No-go error rates and relative EEG beta power per participant

Participant ID	Real or Sham	Left Bar, GO	Right Bar, GO	Left Bar, NOGO	Right Bar, NOGO
1	Real	NE	0.137	-0.287	0.043
1	Sham	NE	NE	NE	-0.110
2	Sham	0.170	-0.093	NE	-0.546
2	Real	NE	-0.036	0.034	NE
3	Real	NE	NE	NE	NE
3	Sham	NE	NE	NE	NE
4	Sham	NE	NE	NE	NE
4	Real	NE	NE	-0.123	NE
5	Real	-0.110	-0.018	0.209	-0.171
5	Sham	NE	NE	NE	-0.147
6	Real	-0.075	0.281	0.196	NE
6	Sham	-0.171	0.313	NE	NE
7	Real	0.088	0.564	NE	0.222
7	Sham	NE	NE	NE	NE
8	Sham	0.145	NE	-0.275	0.003
8	Real	NE	-0.012	0.981	0.332
9	Sham	-0.064	-0.035	NE	-0.154
9	Real	-0.197	0.100	NE	-0.169
10	Real	-0.054	0.041	-0.221	0.173
10	Sham	0.006	-0.115	NE	NE
12	Sham	-0.072	-0.016	NE	NE
12	Real	NE	-0.008	NE	NE
13	Sham	0.268	NE	-0.108	0.873
13	Real	0.127	NE	NE	NE
14	Real	NE	-0.105	-0.187	0.669
14	Sham	NE	-0.151	-0.199	NE
15	Real	NE	-0.128	0.354	-0.180
15	Sham	NE	NE	0.733	-0.434
16	Sham	NE	-0.249	-0.044	-0.622
16	Real	NE	NE	0.031	NE
18	Sham	-0.017	0.013	NE	NE
18	Real	-0.143	0.015	NE	0.050
19	Real	0.270	-0.101	NE	-0.119
19	Sham	-0.171	NE	NE	-0.067
**Average (Real)**		-0.012	0.056	0.099	0.085
**Average (Sham)**		0.010	-0.042	0.021	-0.134

NE = No errors
